# Mitomycin C‐immobilized silver nanoparticle‐loaded polycaprolactone membrane for temporary scalp expansion after decompressive craniectomy to prevent wound infection

**DOI:** 10.1002/btm2.70023

**Published:** 2025-04-30

**Authors:** Kai‐Lun Liu, Ya‐Jyun Liang, Kuo‐Hsiang Hung, Yu‐Ning Chen, Feng‐Huei Lin

**Affiliations:** ^1^ Department of Biomedical Engineering College of Medicine and College of Engineering, National Taiwan University Taipei Taiwan; ^2^ Department of Laboratory Medicine National Taiwan University Hospital Hsin‐Chu Branch Hsinchu Taiwan; ^3^ Division of Neurosurgery, Department of Surgery National Taiwan University Hospital Hsin‐Chu Branch Hsinchu Taiwan; ^4^ Division of Neurosurgery, Department of Surgery National Cheng Kung University Hospital Tainan Taiwan; ^5^ Institute of Biomedical Engineering and Nanomedicine National Health Research Institutes Zhu‐nan Miaoli Taiwan

**Keywords:** AgNPs, artificial scalp, cerebral edema, decompressive craniectomy, PCL

## Abstract

Malignant cerebral edema (MCE) represents a significant medical emergency characterized by unmanageable intracranial pressure (ICP), frequently arising as a consequence of traumatic brain injury (TBI) or ischemic stroke. Decompressive craniectomy (DC) is a prevalent surgical procedure employed to mitigate elevated ICP by excising a segment of the skull to enhance intracranial volume. Nevertheless, in patients suffering from MCE, the limited capacity for expansion of the scalp subsequent to DC may lead to sustained elevated ICP and complications including wound‐edge necrosis, cerebrospinal fluid leakage, and infection. This investigation seeks to formulate a biocompatible, antibacterial, and anti‐adhesive membrane intended for temporary scalp expansion following DC, thereby addressing these pressing concerns. The proposed membrane comprises polycaprolactone (PCL) augmented with silver nanoparticles (AgNPs) to confer antibacterial properties and is further immobilized with Mitomycin C (MMC) to minimize tissue adhesion, thereby facilitating more straightforward removal. The selection of PCL was predicated upon its remarkable mechanical strength and ductility, which make it suitable for withstanding intracranial edema and facilitating the suturing protocol. The AgNPs were synthesized through a green synthesis methodology employing epigallocatechin gallate (EGCG) to ensure environmental sustainability and the stability of the resultant nanoparticles. MMC, known for its anti‐proliferative attributes, was affixed to the PCL surface via oxygen plasma treatment, thereby enhancing the anti‐adhesive properties of the membrane. This study evaluates the mechanical characteristics, antibacterial effectiveness, anti‐adhesive capabilities, and biocompatibility of the PCL/AgNPs/MMC membrane, thereby demonstrating its potential to improve outcomes in DC procedures by increasing intracranial volume and reducing postoperative complications.

**Abbreviations**
AgNPs: silver nanoparticles
CSF: cerebrospinal fluid
DC: decompressive craniectomy
EGCG: epigallocatechin gallate
ICP: intracranial pressure
MCE: malignant cerebral edema
MMC: mitomycin C
PCL: polycaprolactone
TBI: traumatic brain injury

**Translational Impact Statement**

The developed PCL/AgNPs/MMC membrane presents a promising solution for improving outcomes in decompressive craniectomy (DC) by providing temporary scalp expansion, reducing postoperative complications such as infection and tissue adhesion. Its biocompatible, antibacterial, and anti‐adhesive properties facilitate safer and more effective management of malignant cerebral edema (MCE), potentially enhancing patient recovery and surgical success rates. The membrane's innovative design and environmentally sustainable fabrication process highlight its potential for future clinical translation in neurosurgical applications.

## 1 INTRODUCTION

Malignant cerebral edema (MCE) is a fatal medical condition characterized by uncontrollable intracranial pressure (ICP). MCE can result from traumatic brain injury (TBI), ischemic stroke, intracranial infection, intracranial neoplasm, or other brain‐related conditions.^1^ The most common causes of MCE are severe head injury and intracranial large‐vessel occlusion.^2^ The incidence of MCE is estimated to be >10% in patients with severe head injury and between 3% and 10% in patients with anterior circulation ischemic stroke.^2^, ^3^ Without timely intervention, MCE can lead to brain herniation, brainstem damage, and a mortality rate as high as 80%.^1^, ^4^ Decompressive craniectomy (DC) is a surgical procedure performed in patients with medically uncontrollable elevated ICP. This procedure is traditionally tailored to the removal of large areas of bone from the skull, expansion of the dura, and primary closure of the scalp incision. DC can effectively increase intracranial volume and reduce ICP.^1^ However, in patients with MCE, even after DC, ICP may not normalize due to the limited expansion capacity of the scalp. Furthermore, primary closure of the scalp incision containing the severely swollen brain will increase wound tension and cause further complications. These complications include wound‐edge necrosis, dehiscence, cerebrospinal fluid (CSF) leakage, and infection.^2^, ^5^, ^6^ Therefore, to further alleviate ICP and scalp tension in patients with MCE, a temporary artificial scalp that can safely expand the scalp surface area after DC is necessary.^7^ Existing materials for temporary scalp expansion have been associated with high infection rates and CSF leakage, underscoring the need for improved solutions.^2^, ^7^, ^8^, ^9^ This study aims to develop a biocompatible, antibacterial, and anti‐adhesive membrane designed for temporary scalp expansion following DC. The membrane is intended to be sutured to the patient's scalp, thereby increasing intracranial volume and improving survival rates during the critical 10–14‐day period of uncontrollable ICP following brain injury.^10^ After the brain swelling subsides, the artificial scalp can be surgically removed, allowing for wound closure.^7^

In this study, we propose the development of a polycaprolactone (PCL) membrane enhanced with silver nanoparticles (AgNPs) for antibacterial properties and immobilized with Mitomycin C (MMC) to reduce tissue adhesion, facilitating easier future removal. PCL was chosen for this application because of its balanced mechanical strength and flexibility. These properties enable it to handle the pressure from brain swelling and withstand the suturing process without tearing, making it an excellent candidate for temporary artificial scalps.^11^ The mechanical characteristics of PCL, with tensile and elastic moduli ranging from 0.001 to 100 MPa, make it well suited for use in soft tissues like skin, muscle, and cartilage.^12^, ^13^ Moreover, the US Food and Drug Administration (FDA) has already approved PCL for medical purposes.^14^ To boost the antibacterial capabilities of the membrane, AgNPs were embedded into the PCL matrix. These nanoparticles, which consist of 20–15,000 silver atoms and are less than 100 nm in size, have strong antibacterial effects and are effective over long periods, with low chances of bacterial resistance.^15^, ^16^, ^17^ Traditionally, AgNPs are synthesized using chemical reducing agents, which can be harmful to both people and the environment.^18^ In this study, we addressed this concern by using a greener method of synthesis, employing epigallocatechin gallate (EGCG), a plant‐derived polyphenol that acts as both a reducing and stabilizing agent, ensuring the nanoparticles remain stable. In this study, we addressed this concern by using a greener method of synthesis, employing epigallocatechin gallate, a plant‐derived polyphenol that acts as both a reducing and stabilizing agent, ensuring the nanoparticles remain stable.^19^, ^20^, ^21^, ^22^, ^23^ Since the temporary artificial scalp needs to be removed after the brain swelling subsides, minimizing tissue adhesion is crucial to reduce the risk of secondary injury. To achieve this, MMC was incorporated for its anti‐proliferative properties. As an antibiotic, MMC crosslinks with DNA, disrupting cellular activity and effectively preventing tissue adhesion.^23^ To immobilize MMC on the inherently hydrophobic PCL surface, oxygen plasma treatment was employed, rendering the surface hydrophilic and chemically reactive, facilitating drug immobilization.^24^, ^25^

**FIGURE 1:**
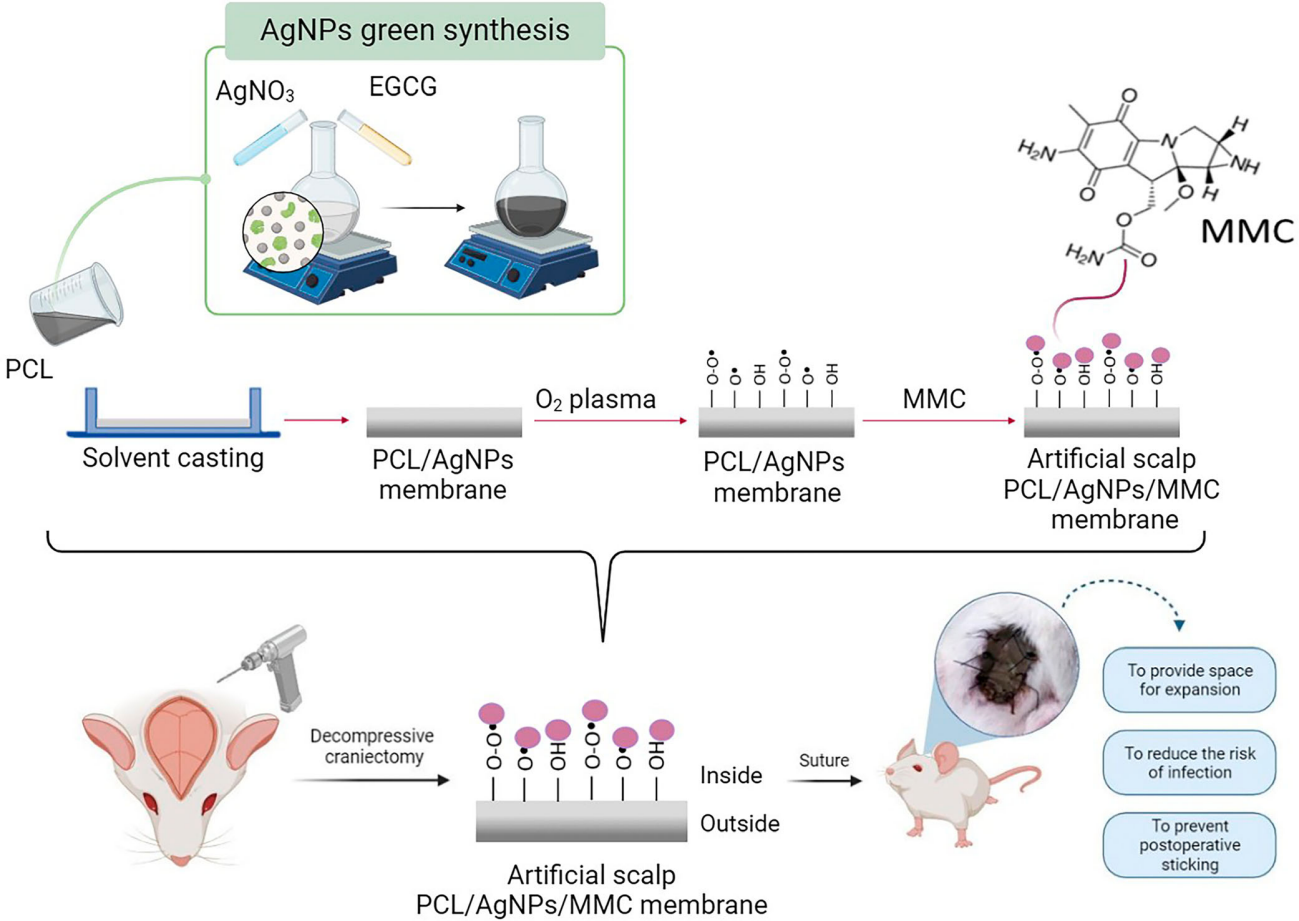
Research purpose and design. To develop a safe and effective temporary artificial scalp made from a PCL and AgNPs membrane with MMC immobilized onto the surface. Designed to join surgically with the patient's scalp, the artificial scalp expands surface area, aiding survival post‐brain surgery. Removed after 2 weeks to preserve more neurological function and minimize infection risk.

This study provides a comprehensive evaluation of the mechanical properties, antibacterial efficacy, anti‐adhesive effects, and biocompatibility of the PCL/AgNPs/MMC membrane for its potential application in DC procedures. The overall design and purpose of this innovative membrane are schematically illustrated in Figure 1, which highlights its potential to increase intracranial volume and reduce the risk of postoperative complications.

## 2 MATERIALS AND METHODS

### 2.1 Preparation of PCL/AgNPs/MMC membrane

AgNPs were synthesized using the EGCG bioreduction method as a green synthesis process.^20^, ^26^ Initially, 0.45 g of AgNO_3_ (Sigma‐Aldrich, USA) was dissolved in 100 mL of deionized water (ddH_2_O) and heated to 65°C while stirring. After 10 min, a solution of 0.27 g of EGCG (TCI, Japan) dissolved in 15 mL of ddH_2_O was gradually added dropwise to the AgNO_3_ solution. After 3 h, the particles were collected, centrifuged at 10,000 rpm for 10 min, and washed with ddH_2_O twice. The precipitated AgNPs were lyophilized.

The PCL/AgNPs membrane was synthesized using the solvent casting method.^27^ The PCL/AgNPs membrane was prepared using 3 wt% PCL in ethyl acetate mixed with 0.25 wt% AgNPs.^28^, ^29^ Initially, 1.5 g of PCL pellets (Mn = 80,000 g/mol, Sigma‐Aldrich, USA) were dissolved in 50 mL of ethyl acetate (Sigma‐Aldrich, USA). The solution was heated and stirred overnight at 50°C to ensure proper mixing and dissolution. Subsequently, 0.25 wt% AgNPs were added to the PCL solution and continuously stirred at 50°C for 30 min to achieve a homogeneous solution. Next, 20 mL of the mixed solution was poured into a circular glass dish with a diameter of 10 cm, where it was allowed to spread evenly and dry for a week. Prior to oxygen plasma treatment, the membrane was vacuum dried for 24 h. The membrane was then placed in a reaction chamber that was pumped down to a vacuum. Subsequently, 200 mTorr of oxygen was introduced, and a power intensity of 30 W was applied for 10 min. The modified membrane was immediately immersed in a 0.5 mg/mL MMC (TCI, Japan) solution to react overnight. The MMC and free radicals or functional groups on the membrane surface bonded and were thereby immobilized. After the reaction, the membrane was washed with ddH_2_O twice and dried in a vacuum oven for 2 days to obtain the PCL/AgNPs/MMC membrane.

### 2.2 Material characterization

The crystalline structure of the AgNPs was determined by x‐ray diffraction (XRD, Rigaku TTRAX 3) operated at a voltage of 45 kV and a current of 20 mA with CuKα radiation (λ = 1.54 nm). The data were recorded in the 2θ range of 10°–70° to confirm the crystalline nature of the AgNPs.^30^ The data were analyzed using MDI JADE 6.5 software to identify the crystal structure. The morphology and particle size of the AgNPs were determined using field‐emission transmission electron microscopy (FE‐TEM, Tecnai G2 F20, Japan). For the TEM analyses, the AgNPs powder was spread on an ultrathin carbon‐coated copper grid and mounted on a holder. The selected‐area electron diffraction (SAED) pattern was examined in high‐resolution TEM (HRTEM) mode. The particle size of the AgNPs was analyzed using dynamic light scattering (DLS, 90Plus/BI‐MAS, USA) at 25°C.^31^ AgNPs were dispersed in distilled ddH_2_O.

The morphology and chemical composition of the PCL/AgNPs/MMC membrane were examined using field‐emission scanning electron microscopy (FE‐SEM, FEI, NOVA NANO SEM 450) and x‐ray energy‐dispersive spectrometry (EDS, Bruker, FQ5060). A properly sized specimen was mounted on the specimen stage using carbon tape, and all samples were sputter‐coated with platinum at 15 mA for 60 s prior to analysis. The morphology and chemical composition of the synthesized material were subsequently observed using SEM and EDS at an accelerated voltage of 10 kV following platinum sputtering.^32^

### 2.3 Measurement of mechanical properties

The mechanical properties, including the tensile strength, Young's modulus, and elongation at break of the PCL/AgNPs/MMC membrane were evaluated according to the ASTM D882‐02 standard method using a tensile testing machine (MTS Landmark, USA).^33^ To perform the tensile test, each sample was precisely cut to a size of 1 × 5 cm^2^ and mounted vertically between two mechanical gripping units of the testing machine with a gauge length of 3 cm. A stress–strain examination was conducted by applying a pulling force to the scaffold at a constant rate of 5 mm/min.^34^ Each sample was tested at least three times to ensure reliable and consistent results. The maximum tensile strength of each sample was recorded and analyzed using the Materials Testing Software. Additionally, the thickness of each sample was measured using an electronic micrometer with a precision of 1 μm. Tensile stress was calculated using Hooke's law, which states that stress (in MPa) is equal to the applied force (*P*) divided by the loaded cross‐sectional area (*A*).^15^

### 2.4 Cytotoxicity of PCL/AgNPs/MMC membrane

The cytotoxicity of the PCL/AgNPs/MMC membrane was assessed using WST‐1 and LDH assays, as well as live/dead staining.^35^ The L929 mouse fibroblast cell line (ATCC) was cultured in Minimum Essential Medium (MEM, Gibco, USA), supplemented with 10% (v/v) fetal bovine serum (FBS, Gibco) and 1% (v/v) penicillin/streptomycin. The cells were maintained at 37°C in a humidified incubator with 5% CO_2_.

For the WST‐1 (Takara, USA) and LDH (Sigma‐Aldrich, USA) assays, L929 cells were seeded in 96‐well plates at a density of 5000 cells/well and incubated for 24 h.^31^ The PCL/AgNPs/MMC membrane extract (6 cm^2^/mL) was prepared using MEM. Zinc diethyldithiocarbamate (ZDEC, Sigma) and aluminum oxide (Al₂O₃, Sigma) at a concentration of 0.2 g/mL served as positive and negative controls, respectively, for 24 hours. Following this period, the medium in the 96‐well plates was replaced with the membrane extract, and the cells were incubated for an additional 24 hours. Cell viability and cytotoxicity were subsequently evaluated using the WST‐1 and LDH assays, with absorbance measured at 450 and 490 nm, respectively, using a SpectraMax iD3 plate reader.

For the live/dead staining (L3224, Invitrogen), L929 cells were seeded in 24‐well plates at a density of 10,000 cells/well and incubated for 24 h.^36^ The PCL/AgNPs/MMC membrane extract was prepared using MEM and applied for 24 h. After incubation, the medium was removed, and the cells were washed with PBS. The cells were then incubated with MEM containing 2 μM calcein‐AM and 4 μM ethidium homodimer‐1 (EthD‐1) for 30 min. Fluorescent staining was visualized using a fluorescence microscope (Olympus FV10000, Japan). Live cells exhibited green fluorescence with excitation/emission maxima of 495/515 nm, while dead cells displayed red fluorescence with excitation/emission maxima of 528/617 nm.

### 2.5 The evaluation of anti‐adhesion

The PCL/AgNPs/MMC membrane features a bilayer structure, with the outer layer composed of PCL/AgNPs for antibacterial functionality and the inner layer composed of PCL/MMC for anti‐adhesive effects. To ensure clarity, we explicitly distinguish between these layers in our experimental procedures.

For cell adhesion assessment, circular membrane samples (diameter: 1.5 cm) were prepared, with coverslips serving as controls. Samples were placed in a 6‐well culture plate, and L929 cells were seeded at a density of 10^5^ cells per well. Since cell adhesion is primarily influenced by the inner PCL/MMC layer, L929 cells were directly seeded onto the membrane surfaces to evaluate the anti‐adhesive effect of MMC.

To visualize cell morphology and attachment, an ActinGreen 488 staining kit (Invitrogen, USA) was used. After 1 day of cultivation, the samples were washed in PBS, fixed in 5% glutaraldehyde solution for 30 min, and permeabilized with 0.1% Triton X‐100 for 10 min at room temperature. Subsequently, ActinGreen reagent was applied, and the samples were incubated in the dark for 30 min. DAPI Fluoromount‐G® (SouthernBiotech, USA) was added to stain cell nuclei for 5 min. Stained cells were observed using a fluorescence microscope (Olympus, FV10000, Japan) at excitation/emission wavelengths of 340/488 nm and 495/518 nm for nuclei and actin, respectively. ImageJ software was used to quantify the percentage of adhered cells per sheet.

Additionally, cell adhesion and morphology were examined via SEM. Following 1 day of cell culture, samples were washed in PBS, fixed in 5% glutaraldehyde solution for 30 min, and dehydrated using an ethanol gradient (30%, 50%, 75%, 80%, 95%, and 100%) for 15 min each. Samples were dried using the critical‐point method, then sputter‐coated with platinum (15 s, 15 mA) before imaging with a Hitachi Tabletop TM‐3000 scanning electron microscope.^37^

### 2.6 The evaluation of anti‐bacterial activity *in vitro*

The antibacterial activity of the PCL/AgNPs/MMC membrane was assessed using the ISO 22196 standard,^38^ which evaluates the antibacterial properties of the outer PCL/AgNPs layer exposed to air. For the antibacterial test, a PCL membrane was used as the control group to compare the antibacterial efficacy of the outer PCL/AgNPs layer. Three bacterial strains, *Escherichia coli* (ATCC 25922), *Pseudomonas aeruginosa* (ATCC 27853), and *Staphylococcus aureus* (ATCC 29213), were selected for the experiment. Colony‐forming units (CFU) were used to quantify the number of viable bacteria.^39^ To assess the anti‐microbial activity, 20 μL of a bacterial solution containing 2 × 10^8^ CFU/mL was applied to circular membranes with a diameter of 3 cm. The membranes were then covered with a 2 × 2 cm film and placed in a sterile Petri dish. After overnight incubation at 35°C, the membranes were washed with 10 mL of thioglycollate broth. Next, 10 μL of the solution was spread on tryptic soy agar (TSA) plates using an L‐shaped stick. The plates were further incubated overnight, and the number of colonies was counted for plates containing 30–300 colonies. The CFU/mL value was calculated using the following formula: CFU/ml = (number of colonies × total dilution factor) ÷ volume of culture placed in mL.

### 2.7 *In vivo* experiment

SD rats were procured from BioLasco Taiwan Co., Ltd., and all animal experiments were performed in accordance with the guidelines of the Institutional Animal Care and Use Committee of the National Taiwan University College of Medicine (IACUC, no. 20220177). After DC, 8‐week‐old male SD rats weighing 250–300 g were randomly divided into three groups, each containing six animals. The three groups included (1) the control group, which received primary scalp closure; (2) the group receiving suturing of a commercial product to the scalp; and (3) the group receiving scalp expansion with a PCL/AgNPs/MMC membrane.

The surgical procedures were performed as follows: The rats were anesthetized using Zoletil (2 mg/100 g, IP) and given meloxicam (0.2 mg/kg, SC) for pain relief. The scalp was shaved and disinfected with 2% w/v Chlorhexidine Gluconate in a 75% v/v alcohol solution. A midline incision was made using Metzenbaum scissors, and the wound size was standardized as 2.5 cm. The skin was then retracted to expose the coronal and lambdoid sutures bilaterally. Craniectomy was performed using a 1.2 mm diamond burr at a speed of 50,000 rpm to drill off the skull bone posterior to the right coronal suture, anterior to the right lambdoid suture, lateral to the sagittal suture, and medial to the right temporalis muscle insertion line. The dura was excised in the craniectomy area. A piece of 5 × 10 mm^2^ artificial dura mater (Biodesign® dura graft, Cook® USA) was placed to facilitate the retrieval of bacterial quantification after the rats were euthanized. Finally, the scalp wounds in the control group were directly closed with continuous 4‐0 Nylon for the sutures. For the two groups undergoing the scalp‐expansion procedure, the wound was closed using a piece of 1 × 2 cm commercial product (commercial‐product group) or 1 × 2 cm PCL/AgNPs/MMC (PCL/AgNPs/MMC group) with interrupted 4‐0 Nylon sutures to the incised scalp.^40^, ^41^ The experiment was conducted over a 2‐week period, and every 3 days, the rats' scalp wounds were carefully examined for signs of infection, necrosis, or CSF leakage and photographed. After 2 weeks, the rats were euthanized, and the artificial dura mater was collected for bacterial quantification. Blood and tissue samples were collected for safety assessments.

### 2.8 Quantitative bacterial culture of the craniectomy area

After the rats were euthanized, the artificial dura mater covering the craniectomy area was collected and placed into an ESwab® collection tube. The tube was then vortexed for 2 min, and 10 μL of the sample was collected and spread evenly onto TSA plates using a sterile L‐shaped rod. The agar plates were then incubated overnight at 35°C. Bacterial colonies were counted and recorded.

### 2.9 Histological analysis

After the rats were euthanized, scalp tissue along the wound was excised using a scalpel. Excess tissue was removed, leaving only the tissue on the edge of the wound for analysis. The specimens were fixed in 10% formaldehyde for a week and subsequently dehydrated by sequential treatment with alcohol (70%–100%) to remove any water. The dehydrated samples were immersed in xylene to remove alcohol and then embedded in paraffin. The paraffin‐embedded samples were cut into 5‐μm sections using a microtome and mounted on glass slides. H&E staining was performed. The stained sections were examined under a light microscope (IX71; Olympus, Tokyo, Japan) and images were captured for histological analysis. The tissue sections were evaluated for the presence of various cellular components, such as inflammatory cells, fibroblasts, and blood vessels, to assess the degree of inflammation and adhesion.^42^

### 2.10 Blood analysis

To assess the safety of the PCL/AgNPs/MMC membranes *in vivo* using CBC and serological analyses, blood samples were collected by cardiac puncture after euthanasia. For serum biochemistry analysis, the samples were allowed to clot for 30 min at room temperature before being centrifuged at 3000 g at 4°C for 15 min to collect the serum.^43^ The analysis included the following parameters: alanine aminotransferase, aspartate aminotransferase, blood urea nitrogen, and creatinine. For the CBC analysis, blood samples were collected in tubes containing ethylenediaminetetraacetic acid. The following parameters were analyzed: red blood cells, hemoglobin, hematocrit, mean cell volume, mean corpuscular hemoglobin, mean corpuscular hemoglobin concentration, reticulocytes, platelets, white blood cells, neutrophils, lymphocytes, monocytes, eosinophils, and basophils. CBC and serological analyses were performed at the National Taiwan University Veterinary Hospital in Taiwan.

### 2.11 Statistical analysis

Statistical data are expressed as mean ± standard deviation (SD). Statistical analyses were performed using a one‐way analysis of variance. Differences were considered significant at a *p*‐value of less than 0.05 (*p < 0.05, **p < 0.01, ***p < 0.001).

## 3 RESULTS

### 3.1 EDS Analysis of AgNPs Distribution

**FIGURE 2:**
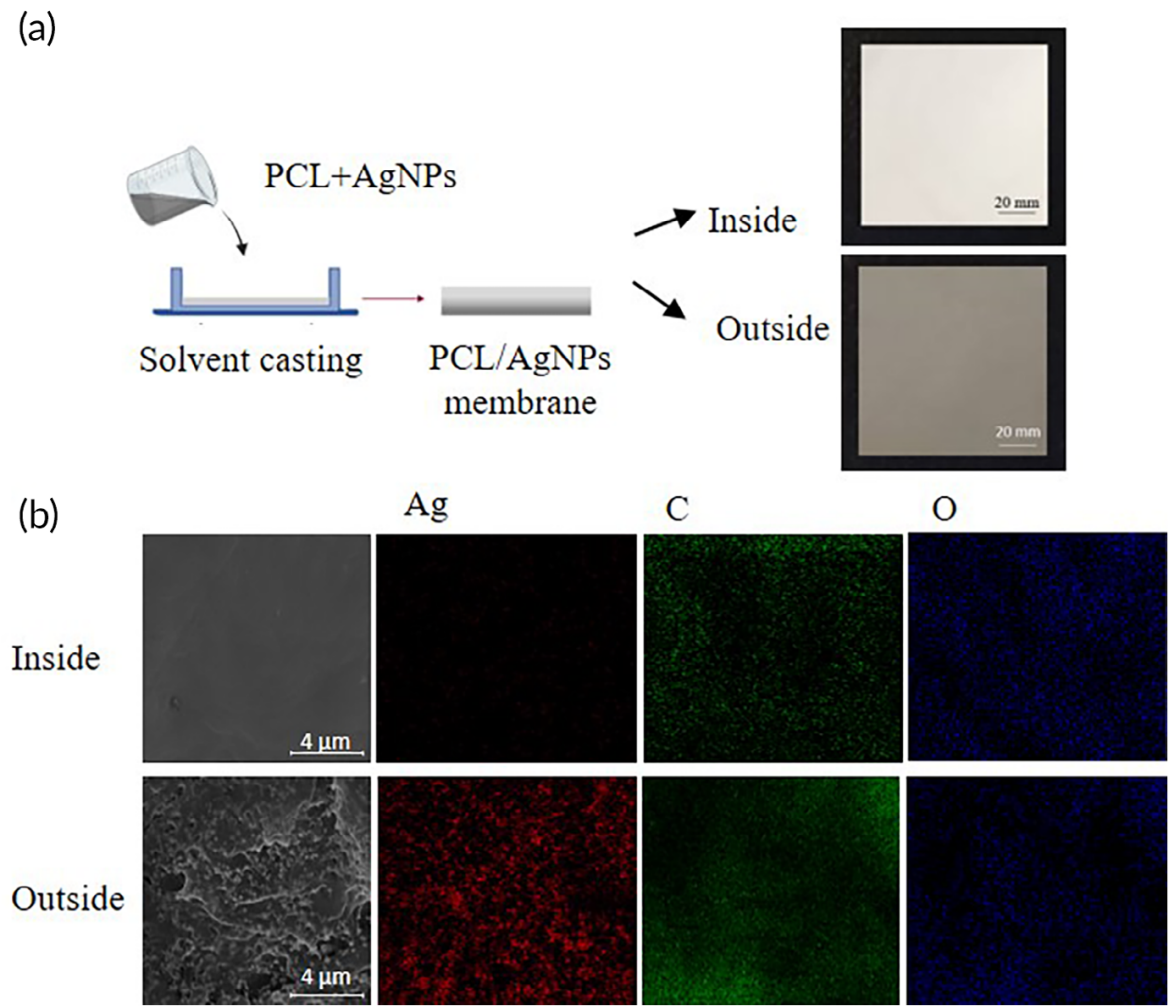
Morphology and chemical composition characterization of PCL/AgNPs membrane. (a) Schematic illusion and photographic image of the PCL/AgNPs membrane fabricated by the solvent casting method. (b) SEM image and EDS elemental mapping images showing the distribution of silver (Ag), carbon (C), and oxygen (O) on the membrane's inner and outer surfaces. These results demonstrate that AgNPs are predominantly concentrated on the outer layer, forming a dense antibacterial surface, while the inner layer remains relatively free of silver. This asymmetric distribution suggests that AgNPs were successfully loaded in a specific orientation, offering a possible approach for antibacterial surface engineering.

In this study, the synthesis of AgNPs demonstrated a crystalline structure with an average size of 86.10 nm, a narrow size distribution (PdI 0.25), and a 10 nm polymer coating, as verified by XRD, TEM, SAED, and DLS analysis. This proved the efficacy of EGCG as a green reductant and stabilizer, as shown in Figure S1. AgNPs were incorporated into PCL via solvent casting to produce a persistent antibacterial membrane, with the side in contact with the head designated as the inner layer and the side exposed to air as the outer layer, which required antibacterial properties. The membrane's appearance, as shown in Figure 2a, reveals a white inner layer and a gray‐black outer layer, likely due to the physical sedimentation of AgNPs, resulting in natural stratification and the formation of two distinct layers. The chemical composition of the synthesized PCL/AgNPs membrane was further analyzed using EDS coupled with SEM, as shown in Figure 2b. SEM images confirmed the presence of numerous AgNPs distributed across the outer layer, while the inner layer remained free of AgNPs, suggesting that the outer layer provides the desired antibacterial properties. The EDS mapping analysis showed the mapping of silver (Ag), carbon (C) and oxygen (O) on the surface of the PCL/AgNPs inside/outside membrane. PCL is an organic compound, mainly composed of C and O elements. Since AgNPs are mainly deposited on the outer membrane, in addition to the elemental distribution of C and O, a distinct Ag signal distribution can also be observed. Such a result may help to provide good antibacterial potential on the outer layer.

### 3.2 Morphology and chemical composition characterization of PCL/AgNPs/MMC membrane

**FIGURE 3:**
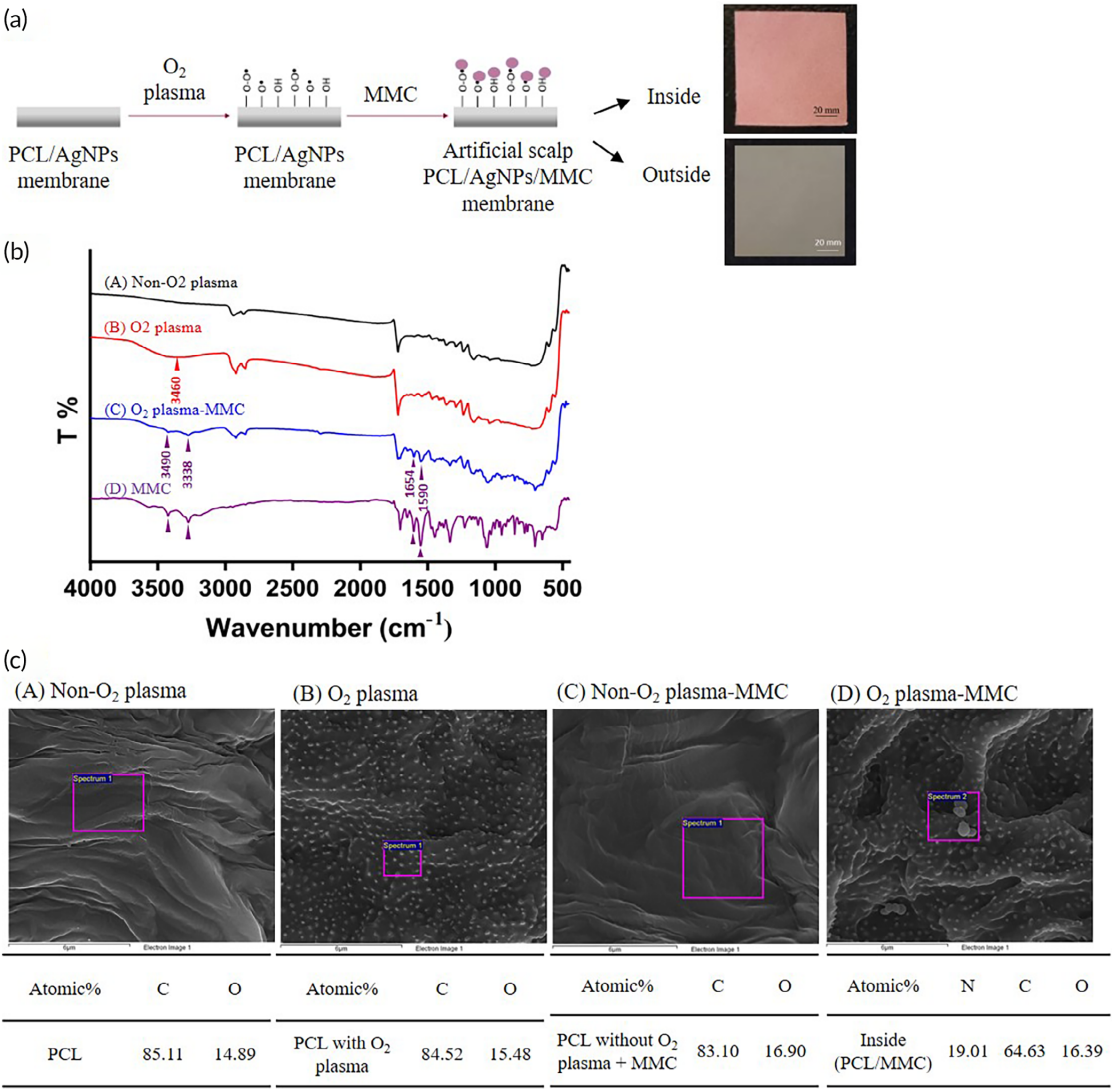
Morphology, chemical composition, and functional group characterization of PCL/AgNPs/MMC membrane. (a) Schematic representation of the fabricated PCL/AgNPs/MMC membrane using O₂ plasma surface modification method. (b) FTIR analysis confirmed successful immobilization of mitomycin C (MMC) on the membrane surface. Characteristic absorption peaks at 3460, 3490, 3338, 1654, and 1590 cm^−1^ correspond to functional groups associated with both MMC and PCL, indicating covalent bonding and molecular interactions.^44^ (c) EDS spectra revealed the presence of a nitrogen (N) signal only in the _O₂_ plasma‐treated MMC (group D), with a notable atomic ratio of 19.01%, suggesting successful MMC incorporation. In contrast, groups A, B, and C (without O₂ plasma activation or MMC treatment) exhibited no nitrogen (N) signals, verifying the specificity and effectiveness of the surface modification process.

In the studies presented, PCL/AgNPs membranes were successfully synthesized and modified using oxygen plasma treatment (Figure S2), followed by immediate immersion in a 0.5 mg/mL MMC solution for 1 day. The selection of the 0.5 mg/mL MMC concentration was based on preliminary experiments, in which membranes were immersed in MMC solutions of 0.3, 0.5, and 1.0 mg/mL, with the drug loading efficiency (EE%) calculated. The 0.5 mg/mL condition achieved the highest EE% of 44.18% ± 0.3% (Table S1) and was thus chosen as the optimal concentration. Notably, the inner membrane's appearance changed from beige to purplish‐pink, likely due to MMC incorporation, while the outer membrane remained gray‐black (Figure 3a). FTIR analysis confirmed the successful immobilization of MMC onto the PCL/AgNPs membrane, as evidenced by characteristic absorption bands. Specifically, the O–H bonds related to oxygen plasma were indicated by an absorption band at 3460 cm^−1^, while the absorption bands at 3490, 3338, 1654, and 1590 cm^−1^ correspond to the O–H and C‐N bonds of MMC (Figure 3b).^44^, ^45^, ^46^ The presence of these characteristic bands in the spectra confirmed the effective immobilization of MMC onto the PCL membrane. MMC, an antibiotic, is composed of C, H, O, and N elements. Chemical composition analysis shows a significant nitrogen (N) signal, with an atomic ratio of 19.01%, in the O_2_ plasma‐PCL/MMC membrane, which is absent in the other groups (Figure 3c, D). Furthermore, a comparison was conducted between PCL membranes before and after oxygen plasma treatment, and PCL membranes directly immersed in MMC solution without oxygen plasma treatment, as shown in Figure 3c, A–C, further validated the successful modification of the PCL membrane with oxygen plasma and the efficient immobilization of MMC.

### 3.3 Mechanical measurement

**FIGURE 4:**
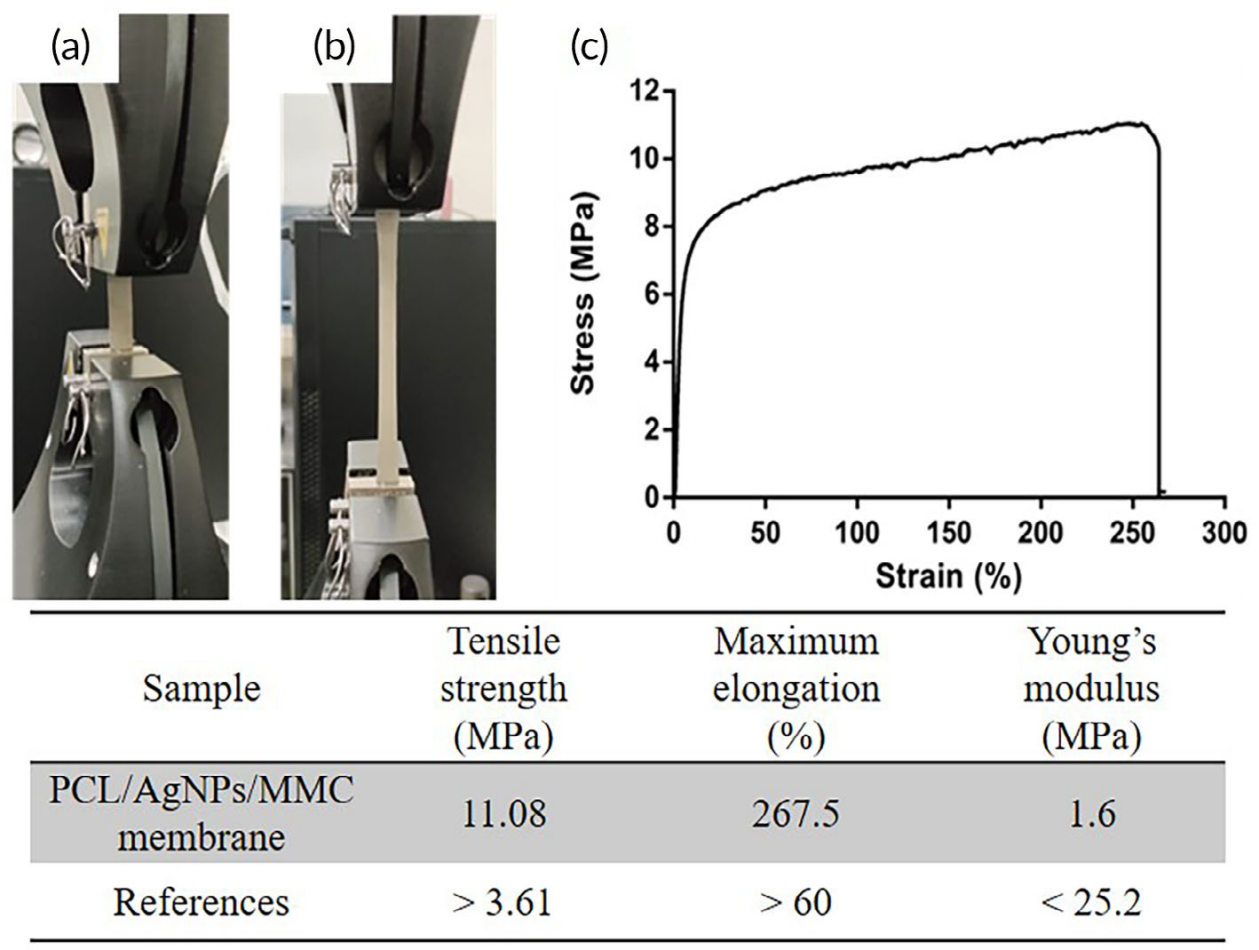
Tensile test of PCL/AgNPs/MMC membrane. The membranes were pulled at 5 mm/min to conduct stress–strain analyses. Each sample was analyzed at least three times. Photographs of PCL/AgNPs/MMC membrane (a) before and (b) after stretching. (c) Stress–strain curve of PCL/AgNPs/MMC membrane. The PCL/AgNPs/MMC membrane exhibits a tensile strength of 11.8 MPa—significantly higher than scalp tissue (3.61 MPa)—with a maximum elongation at break of 267.5%, indicating excellent ductility, while its low Young's modulus of 1.6 MPa suggests susceptibility to deformation under external forces, meeting anticipated mechanical performance criteria.^47^

The tensile stress–strain curves of the PCL/AgNPs/MMC membrane are illustrated in Figure 4, and the corresponding mechanical properties are summarized in the Table. The tensile strength of the PCL/AgNPs/MMC membrane was 11.8 MPa, significantly higher than the general tensile strength of the scalp (3.61 MPa). The membrane exhibited a maximum elongation at break of 267.5%, demonstrating excellent ductility. The Young's modulus of the PCL/AgNPs/MMC membrane, calculated from the elastic region of the stress–strain curves, was 1.6 MPa. This low Young's modulus indicates a high susceptibility to deformation under external forces. These findings suggest that the PCL/AgNPs/MMC membrane possesses favorable mechanical properties and elongation, meeting our anticipated performance criteria.

### 3.4 Cell viability, cytotoxicity, and cell anti‐adhesion of PCL/AgNPs/MMC membrane

**FIGURE 5:**
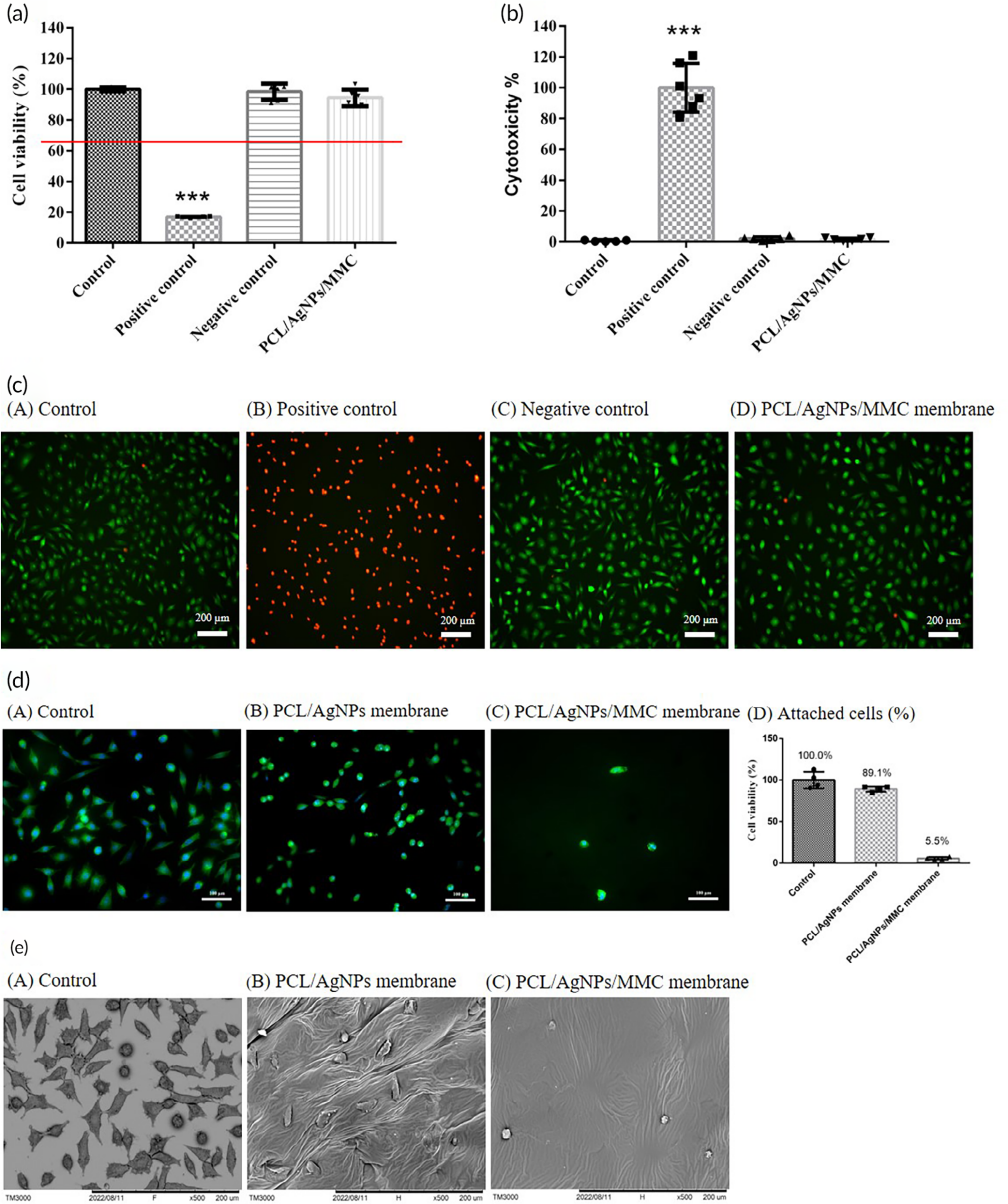
*In vitro* biocompatibility and anti‐adhesion assessment of PCL/AgNPs/MMC membranes using L929 fibroblasts. (a) Cell viability was analyzed using the WST‐1 assay in accordance with ISO‐10993 guidelines. (b) Cytotoxicity was evaluated via LDH release, and the results showed minimal cell damage (***p < 0.001 compared with the control group). (c) Live/dead staining using calcein AM (green, viable cells) and ethidium homodimer‐1 (red, dead cells) confirmed the membrane has no cytotoxic effect. (d) Cell anti‐adhesion properties were assessed by F‐actin (green) and nucleus staining (blue) fluorescence staining. L929 fibroblasts on PCL/AgNPs/MMC membranes displayed a rounded morphology with significantly lower cell attachment (5.5%) compared to the fully distributed and flattened morphology in the control group (100%). Scale bars = 100 μm; original magnification of 200×. (e) SEM images of cell adhesion after 24 h of incubation. Group (A): control group (coverslip), Group (B): PCL/AgNPs membrane, and Group (C): PCL/AgNPs/MMC membrane. The PCL/AgNPs/MMC group exhibited sparse and rounded cell morphology, consistent with poor adhesion. Scale bar = 200 μm; original magnification of 500x.

To assess the biocompatibility of the PCL/AgNPs/MMC membrane, WST‐1, LDH, and live/dead staining experiments were conducted using L929 cells (Figure 5). According to ISO 10993 standards, a material is considered non‐cytotoxic if cell viability exceeds 75% in the WST‐1 analysis (Figure 5a) and cellular toxicity is below 20% in the LDH assay (Figure 5b). In this study, the PCL/AgNPs/MMC membrane met these criteria. Subsequently, live/dead staining was observed under a fluorescence microscope. In the control and negative control groups, cells exhibited healthy green fluorescence, indicating robust growth, whereas the positive control group treated with the ZDEC extract displayed a considerable number of dead cells. Furthermore, the experimental group treated with extracts containing the PCL/AgNPs/MMC membrane showed cell growth with no dead cells (Figure 5c). Based on these findings, it was concluded that the developed PCL/AgNPs/MMC membrane exhibits excellent biocompatibility.

The cell anti‐adhesion of PCL/AgNPs/MMC membrane was observed via F‐actin and nucleus staining (Figure 5d) and SEM (Figure 5e). The term “PCL/AgNPs/MMC membrane” refers to the final composite membrane used in the experiment, which consists of an outer antibacterial PCL/AgNPs layer and an inner anti‐adhesive PCL/MMC layer. Although the anti‐adhesive experiment specifically evaluated the inner PCL/MMC layer, we refer to this experimental group as the “PCL/AgNPs/MMC membrane” in the results to emphasize that all experiments were conducted using the complete composite membrane. The results indicated that cells adhered to the control group exhibited a well‐spread and flattened morphology 24 h post‐incubation, whereas cells on the PCL/AgNPs/MMC membrane displayed a more rounded morphology. Quantitative analysis of cell attachment revealed that the number of cells on the control membranes was comparable to that on the PCL/AgNPs membranes, while the PCL/AgNPs/MMC membranes exhibited a significantly reduced cell attachment rate (100% vs. 5.5%). Furthermore, cell attachment to the PCL/AgNPs membranes was observed to be slightly lower than in the control group (100% vs. 89.1%). These results suggest that the immobilization of MMC on the membrane, rather than the presence of AgNPs, is primarily responsible for the anti‐adhesive properties observed in the PCL/AgNPs/MMC membrane. SEM analysis further corroborated these findings, showing that L929 fibroblasts did not adhere to the surface of the PCL/AgNPs/MMC membrane, in contrast to the other groups (Figure 5e). Based on the combined data from fluorescence microscopy and SEM, it can be concluded that the synthesized artificial scalp, represented by the PCL/AgNPs/MMC membrane, effectively inhibits L929 fibroblast adhesion.

### 3.5 The evaluation of anti‐bacterial activity *in vitro*

**FIGURE 6:**
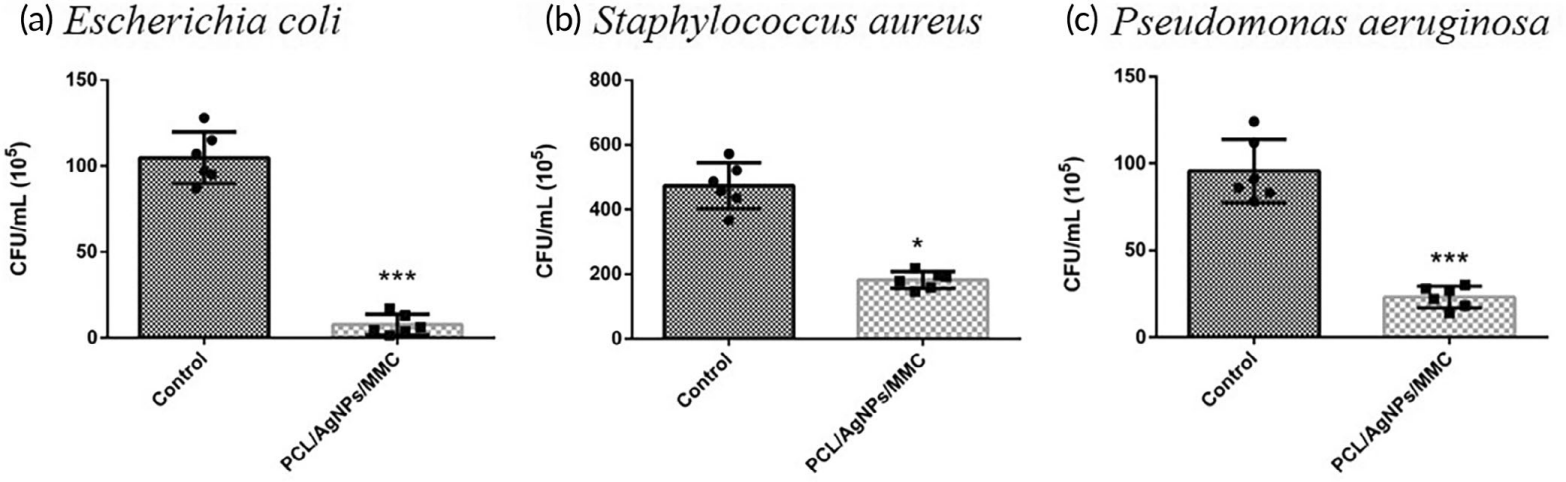
Antibacterial efficacyof PCL/AgNPs/MMC membrane against common pathogenic bacterial. The antibacterial efficacy of the PCL/AgNPs/MMC membrane was evaluated against (a) *Escherichia coli (E. coli)*, (b) *Staphylococcus aureus (S. aureus)*, and (c) *Pseudomonas aeruginosa (P. aeruginosa)*. Quantitative antibacterial assays demonstrated significant reductions in bacterial viability compared to the control group, PCL/AgNPs/MMC membrane significantly inhibition rates of 92.7% for *E. coli*, 61.5% for *S. aureus*, and 75.7% for P. aeruginosa (***p < 0.001 for all comparisons). These results confirm the strong antibacterial properties of PCL/AgNPs/MMC membrane, attributed to silver nanoparticles.

The “PCL/AgNPs/MMC membrane” refers to the final composite membrane with an outer antibacterial PCL/AgNPs layer and an inner anti‐adhesive PCL/MMC layer. The antibacterial experiment specifically evaluated the outer PCL/AgNPs layer; however, to indicate that all experiments were conducted using the complete composite membrane, we refer to this experimental group as the “PCL/AgNPs/MMC membrane” in the results. Figure 6a–c illustrate that the PCL/AgNPs/MMC membrane, compared to the control group, reduced bacterial growth by 92.7% for Escherichia coli, 61.5% for Staphylococcus aureus, and 75.7% for *P. aeruginosa*. These findings demonstrate the PCL/AgNPs/MMC membrane's significant antibacterial efficacy in accordance with the ISO 22196 standard.

### 3.6 *In vivo* experiment

**FIGURE 7:**
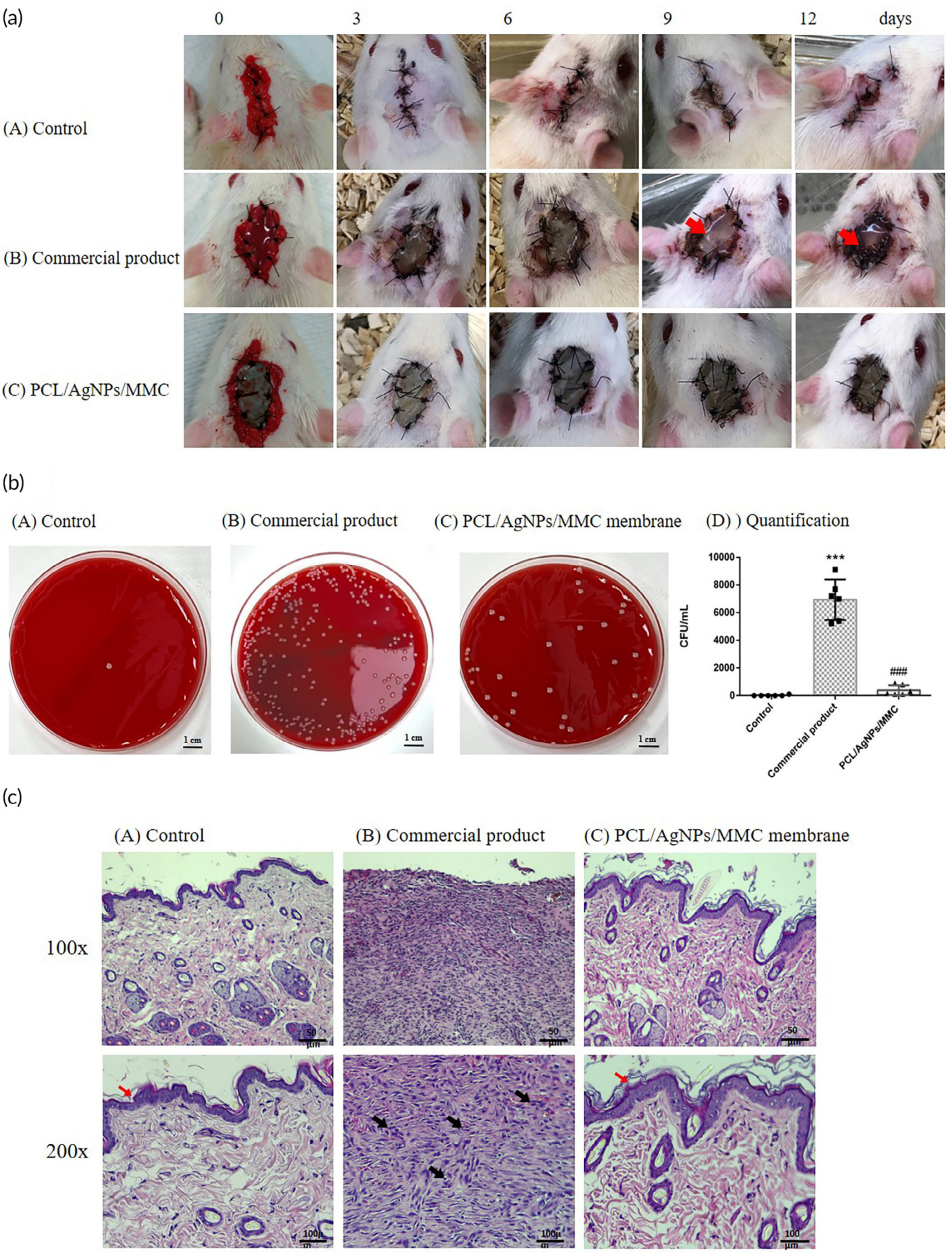
*In vivo* evaluation of wound healing and antibacterial efficacy of PCL/AgNPs/MMC membrane in a rat decompressive craniectomy model. (a) Representative postoperative wound images captured on days 0, 3, 6, 9, and 12 in male Sprague‐Dawley rats treated with direct scalp suturing (control), a commercial wound dressing, or the PCL/AgNPs/MMC. All animals survived without neurological deficits. Notably, the PCL/AgNPs/MMC membrane group exhibited significantly reduced wound exudate and better wound appearance compared to the commercial product, indicating superior biocompatibility and wound management potential. (b) Quantitative *in vivo* antibacterial analysis of the craniotomy site. CFU/mL quantification shows significantly fewer colonies in the PCL/AgNPs/MMC group (***p < 0.001 vs. control; ###*p* < 0.001 vs. commercial product), demonstrating superior antibacterial properties. (c) Histopathological assessment of the wound healing at day 14 post‐surgery. H&E staining of scalp wound tissue revealed minimal inflammatory infiltration and tissue adhesion in the PCL/AgNPs/MMC group. Well‐organized dermal architecture, collagen deposition, and hair follicle regeneration were observed, comparable to the commercial product group. Red arrows indicate epithelium and cutin layers; black arrows highlight inflammatory cell accumulation. Scale bars: 50 μm (100×), 100 μm (200×)

The results showed that all three groups of rats survived with no neurological deficits. The wound conditions for each group are shown in Figure 7a. The wounds in the commercial‐product group exhibited significantly more exudate than those in the PCL/AgNPs/MMC group, potentially increasing infection risk. In contrast, both the control and PCL/AgNPs/MMC groups showed less exudate and perifocal erythema, suggesting that scalp expansion with the PCL/AgNPs/MMC membrane may be safer and more effective.

Bacterial cultures of the craniectomy area (Figure 7b) revealed that 2 weeks post‐surgery, the PCL/AgNPs/MMC group had significantly fewer bacterial colonies than the commercial‐product group. Figure 7b, D highlights notable differences in bacterial growth between the control and commercial‐product groups, as well as between the commercial‐product and PCL/AgNPs/MMC groups, confirming the membrane's superior antibacterial properties.

Histological analysis (Figure 7c) further supports these findings. H&E staining showed that 14 days post‐surgery, the commercial‐product group had increased inflammatory cell and fibroblast aggregation (black arrows), indicating more severe inflammation and adhesion. In contrast, the PCL/AgNPs/MMC group exhibited normal dermal and epidermal structure, abundant collagen deposition, and visible hair follicles, similar to the control group. These results demonstrate that the PCL/AgNPs/MMC membrane is a superior material for temporary scalp expansion.

## 4 DISCUSSION

The development of the PCL/AgNPs/MMC membrane addresses critical challenges in temporary scalp expansion following DC by combining antibacterial and anti‐adhesive properties with mechanical robustness and biocompatibility. Traditional nanoparticle synthesis methods often rely on chemical reducing agents like sodium borohydride and hydrazine, which pose environmental and health risks.^48^ In this study, we adopted a green synthesis approach, using EGCG as both a reducing and stabilizing agent to produce AgNPs within a PCL matrix. FE‐TEM analysis revealed that the synthesized AgNPs exhibited an irregular spherical shape and were coated with a layer of amorphous polymer with a thickness of approximately 10 nm (Figure S1). This layer is believed to contribute to AgNPs stabilization.^21^, ^49^, ^50^ This eco‐friendly method not only adheres to green chemistry principles but also ensures high nanoparticle stability and prevents agglomeration, yielding AgNPs with an average size of approximately 86 nm.^49^ The antibacterial efficacy of AgNPs is well‐documented, primarily through two mechanisms. First, their nanoscale size facilitates enhanced penetration into bacterial cells, allowing interaction with DNA and proteins, leading to membrane destabilization, inhibition of DNA replication, and suppression of bacterial proliferation. Second, AgNPs can induce the generation of reactive oxygen species (ROS), which cause oxidative stress, resulting in bacterial apoptosis and cell lysis.^51^, ^52^ *E. coli, P. aeruginosa*, and *S. aureus* are the most common pathogens associated with wound infections.^16^ Silver ions at a concentration of approximately 1 μg/mL have been shown to inhibit the growth of *Escherichia coli, Staphylococcus, Providence, Serratia*, and *P. aeruginosa*. Silver‐containing wound dressings have demonstrated antibacterial properties against methicillin‐resistant *Staphylococcus aureus* (MRSA).^53^ Our results confirm the PCL/AgNPs/MMC membrane's significant antibacterial activity, particularly against *E. coli*, *P. aeruginosa*, and *S. aureus*, which are common pathogens in wound infections. The membrane reduced bacterial proliferation by 92.7% for *E. coli*, 61.5% for *S. aureus*, and 75.7% for *P. aeruginosa*, demonstrating its potential to reduce infection rates in clinical settings (Figure 6).

The double‐layered structure of the PCL/AgNPs membrane was formed by the natural settling of the AgNPs particles (Figure 2). During membrane production, the bottom layer, designed to be the outer layer during the scalp‐expansion procedure, had a higher concentration of AgNPs. The other layer, designated as the inner layer, was immobilized with MMC. Oxygen plasma treatment can break covalent bonds, resulting in free radicals and highly reactive functional groups.^37^, ^54^ This induces chemical alterations and enhances the presence of oxygen‐containing functional groups on the surface.^55^ Consequently, the hydrophobic surface of PCL was transformed into a hydrophilic and chemically reactive surface, facilitating its interaction with the hydrophilic MMC (Figure 3 and S2). To further investigate the chemical modifications induced by plasma treatment and confirm the successful immobilization of MMC, we performed XPS analysis (Figure S3). The carbon spectra revealed the formation of C=O functional groups after oxygen plasma treatment, indicating successful surface activation. Additionally, in the MMC‐loaded PCL membrane, the presence of additional amide bond functional groups was observed, further verifying MMC immobilization. The bonding mechanism involves the formation of amide bonds, where free radicals generated during plasma treatment rapidly react with plasma species, leading to the formation of polar functional groups such as C=O, O=C‐O, C‐N, C=N, and N‐C=O.^37^, ^56^, ^57^ This double‐layer structure was designed to fulfill two requirements: antibacterial properties in the outer layer, which is exposed to the external environment, and anti‐adhesive properties in the inner layer, thus facilitating easier removal of the artificial scalp later without causing secondary injuries or removal difficulties. Based on these results, we successfully synthesized a double‐layered PCL/AgNPs/MMC membrane. This dual functionality is critical for preventing infections and minimizing tissue adhesion during the postoperative period.

According to mechanical strength data of the human scalp, an artificial scalp should exhibit a tensile strength exceeding 3.6 MPa to withstand rupture during brain swelling, while maintaining a tensile modulus below 25.20 MPa to ensure adequate elasticity.^34^ Mechanical testing confirmed that the PCL/AgNPs/MMC membrane possesses a tensile strength of 11.08 MPa and a maximum elongation of 267.5%, surpassing the mechanical requirements for human scalp tissue. Its Young's modulus of 1.6 MPa indicates sufficient flexibility to accommodate intracranial swelling without rupturing (Figure 4). These mechanical properties ensure that the membrane can withstand the pressures associated with brain swelling while maintaining its integrity.

Biocompatibility tests, in accordance with ISO 10993 regulations, demonstrated that the PCL/AgNPs/MMC membrane is safe for *in vitro* applications, with no adverse effects on cell viability (Figure 5a–c). The anti‐adhesive properties provided by MMC further enhance the membrane's functionality by reducing the risk of postoperative tissue adhesion, which can complicate subsequent removal. Importantly, the presence of AgNPs did not negatively impact cell viability, underscoring the membrane's safety for clinical use (Figure 5d,e).

*In vivo* experiments reinforced the membrane's potential for clinical application. The PCL/AgNPs/MMC membrane demonstrated the necessary strength and flexibility to endure 4‐0 Nylon sutures without tearing or causing CSF fistula formation. While the collagen components of commercially available artificial skin products can retain moisture in wounds to enhance skin growth,^58^, ^59^ this moisture retention can increase the infection rate in temporary scalp‐expansion procedures.^58^, ^60^ Compared to commercial artificial skin products, our membrane exhibited less wound exudate, fewer bacterial colonies, and reduced inflammation and fibroblast activity, indicating superior performance in preventing infections and minimizing tissue adhesion (Figure 7). Additionally, CBC and serological analyses confirmed that the membrane did not trigger systemic inflammation or hepatorenal toxicity, further validating its biocompatibility (Tables S2 and S3).

Overall, the PCL/AgNPs/MMC membrane developed in this study represents a promising solution for improving outcomes in patients undergoing DC by providing enhanced antibacterial protection and reducing tissue adhesion, thereby mitigating common postoperative complications. Its dual functionality, combined with mechanical durability and biocompatibility, makes it a promising alternative to existing commercial products.

## 5 CONCLUSION

This study developed a bi‐layer PCL/AgNPs/MMC membrane for temporary scalp expansion after decompressive craniectomy. AgNPs, synthesized via green reduction using EGCG, were integrated into the PCL matrix, while MMC was immobilized via oxygen plasma treatment, endowing the membrane with antibacterial and anti‐adhesive properties. The membrane exhibited high mechanical strength, with a tensile strength of 11.08 MPa and a maximum elongation at break of 267.5%, ensuring sufficient flexibility for scalp expansion procedures. Biocompatibility assessments, including WST‐1, LDH, and live/dead staining assays, confirmed compliance with ISO10993 standards. Antibacterial evaluations demonstrated significant bacterial inhibition, reducing growth by 92.7% for *E. coli*, 61.5% for *S. aureus*, and 75.7% for *P. aeruginosa*. The membrane also exhibited strong anti‐adhesive properties, inhibiting cell adhesion by 94.5%, which facilitates easier removal after temporary use. *In vivo* experiments confirmed its efficacy, showing reduced bacterial colonization, improved wound healing, and minimal inflammation compared to commercial products, with no systemic toxicity. In conclusion, the PCL/AgNPs/MMC membrane presents a promising solution for clinical application in temporary scalp expansion, offering enhanced safety and effectiveness in managing severe cerebral edema post‐decompressive craniectomy. Its combination of antibacterial, anti‐adhesive, and favorable mechanical properties positions it as a valuable advancement in the field of biomedical materials for neurosurgical applications.

## AUTHOR CONTRIBUTIONS

**Kai‐Lun Liu**: Conceptualization, formal analysis, investigation, methodology, data analysis, visualization, writing—original draft. **Ya‐Jyun Liang**: Conceptualization, investigation, methodology, visualization, writing—review and editing. **Kuo‐Hsiang Hung**: Methodology, data analysis. **Yu‐Ning Chen**: Conceptualization, writing—review and editing, funding acquisition, supervision. **Feng‐Huei Lin**: Conceptualization, writing—review and editing, funding acquisition, supervision.

## FUNDING INFORMATION

This study was supported by the National Health Research Institutes, Taiwan (BN‐113‐PP‐01 and BN‐113‐GP‐09), and the National Taiwan University Hospital Hsin‐Chu Branch (112‐HCH040).

## CONFLICT OF INTEREST STATEMENT

The authors declare no conflict of interest.

## Supporting information


**Data S1:** Supporting Information

## Data Availability

The data that support the findings of this study are available on request from the corresponding author. The data are not publicly available due to privacy or ethical restrictions.
