# Supplementary material for: Mitomycin C‐immobilized silver nanoparticle‐loaded polycaprolactone membrane for temporary scalp expansion after decompressive craniectomy to prevent wound infection
Source: Bioeng Transl Med. 2025 Apr 30;10(5):e70023. doi: 10.1002/btm2.70023 (PMC12478333; doi:10.1002/btm2.70023)
Supplement: Supplementary file 1 — Data S1: Supporting Information [file BTM2-10-e70023-s001.docx]

AgNPs Characterizations

The XRD patterns was used for the analysis of the crystal structure of the synthesized AgNPs in the study. Figure 2a is the XRD diffraction pattern of synthesized AgNPs; where all the characteristic peaks such as (200), (111), and (220) are fully matched to the standard pattern of AgNPs as a JCPD card of 04-0783. These results confirmed that the synthesized AgNPs possess the same crystal structure as previously determined. TEM analysis further revealed that the AgNPs possessed an irregular spherical shape, coated with an approximately 10 nm thick amorphous polymer layer (Figure 2b). The SAED patterns indicated that the AgNPs are crystalline, with bright concentric diffraction rings, signifying the presence of (111), (200), and (220) planes of the face-centered cubic (fcc) silver nanoparticles, as shown in Figure S1(c). This corroborates the XRD results, confirming the structural integrity of the AgNPs. The average particle size and distribution of AgNPs analyzed by DLS are shown in Figure S1(d). The average particle size of AgNPs is 86.10 ± 1.60 nm; and PdI is 0.25 ± 0.00. The narrow hydrodynamic size distribution indicated that the prepared AgNPs exhibited a concentrated size range. These findings demonstrate the effective role of EGCG as a green reductant and stabilizer in the synthesis of AgNPs.


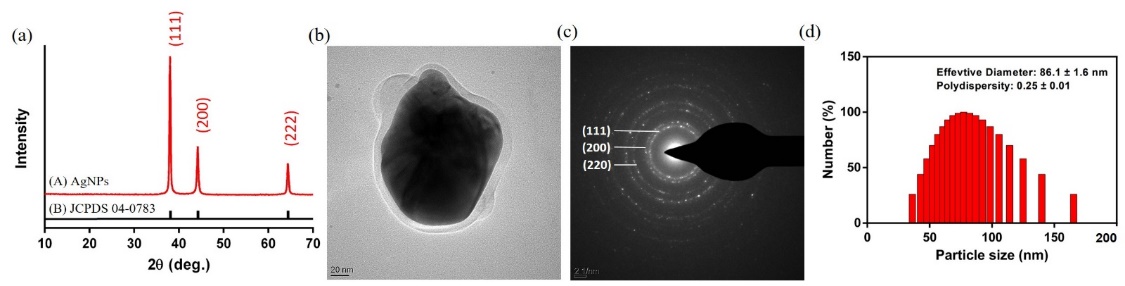


Figure S1 AgNPs Characterizations. (a)The XRD pattern of AgNPs. XRD patterns (111), (200), and (222) for AgNPs. Matching with JCPDS Card No. 04-0783 confirms silver crystallinity. (b) TEM images exhibited an irregular spherical shape of AgNPs. (c) The TEM electron diffraction pattern of the AgNPs show the lattice planes (111), (200), and (220). (d) The corresponding diameter distributions of the AgNPs showed a narrow hydrodynamic size distribution, indicating that the prepared AgNPs exhibited a concentrated size range.


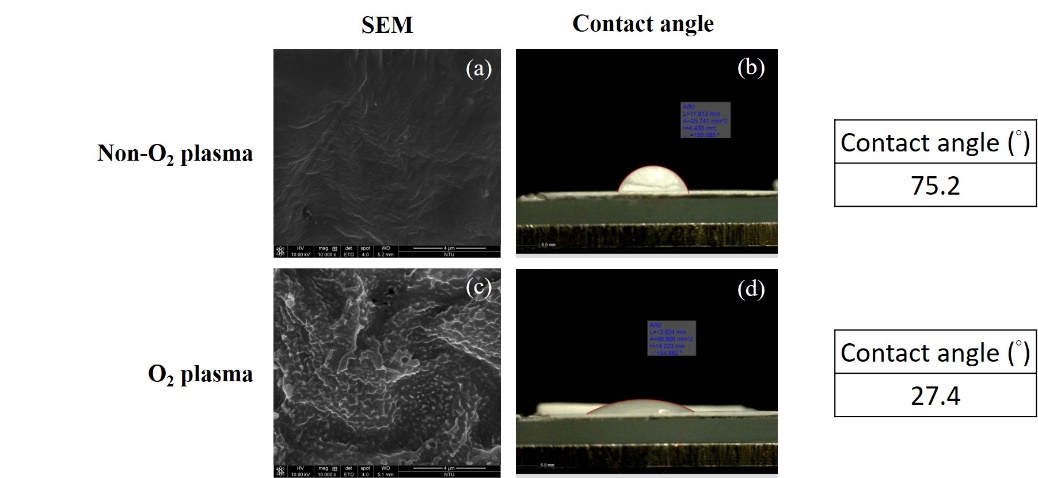


Figure S2 The contact angle of the PCL/AgNPs inside membrane. FE-SEM images and contact angle of inside membrane (a, b) without oxygen plasma and (c, d) with oxygen plasma.


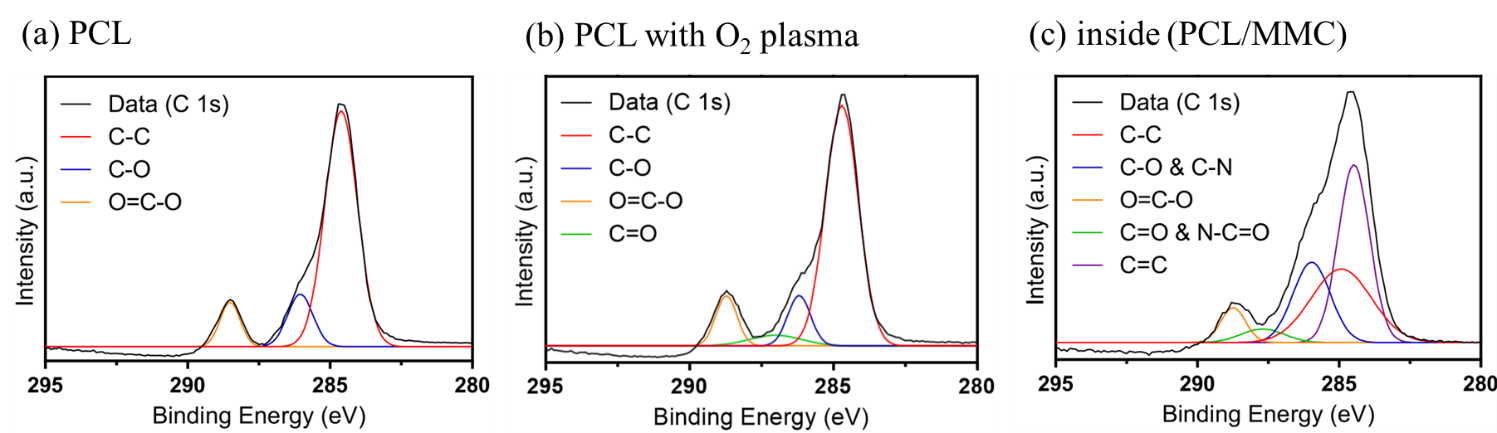


Figure S3 High resolution deconvoluted XPS peaks of (a) PCL, (b) PCL with O2 plasma, and (c) inside (PCL/MMC).

Table S1. The encapsulation efficiency at different MMC concentration were analysis by UV–VIS spectrophotometer at a wavelength of 358 nm.

|  | **MMC**  **concentration** | **EE%** |
| --- | --- | --- |
| **PCL/AgNPs membrane**  **(1 cm^2^)** | 0.3 mg/ml | 39.75 ± 0.2% |
|  | 0.5 mg/ml | 44.18 ± 0.3% |
|  | 1.0 mg/ml | 12.79 ± 0.5% |

$$Encapsulation efficiency (EE\%)= \frac{amount of MMC encapsulated}{total amount of MMC}\times\text{100\%}$$

Table S2. Blood biochemical analysis. Blood biochemical analysis of the serum concentration of aspartate aminotransferase (ALT), alanine aminotransferase (AST), blood urea nitrogen (BUN), creatinine (Crea). Reference: Charles River Laboratories, CD^®^ IGS Rat Model Information Sheet [38].

|  | Control | Commercial product | PCL/AgNPs/MMC membrane | Reference |
| --- | --- | --- | --- | --- |
| **ALT (U/L)** | 51.25 ± 10.04 | 56.60 ± 6.48 | 57.00 ± 18.76 | 65.01 ± 32.28 |
| **AST (U/L)** | 98.23 ± 14.19 | 97.38 ± 7.51 | 93.67 ± 17.87 | 113.67 ± 64.46 |
| **BUN (mg/L)** | 17.05 ± 3.26 | 18.06 ± 2.12 | 16.97 ± 1.33 | 14.19 ± 4.29 |
| **Crea (mg/dL)** | 0.56 ± 0.08 | 0.50 ± 0.11 | 0.53 ± 0.11 | 0.45 ± 0.11 |

Table S3. Safety of PCL/AgNPs/MMC membrane in vivo by blood element analysis. Reference: Charles River Laboratories, CD^®^ IGS Rat Model Information Sheet.

|  | Control | Commercial product | PCL/AgNPs/MMC membrane | Reference |
| --- | --- | --- | --- | --- |
| **RBC (M/µL)** | 8.19 ± 0.18 | 7.72 ± 0.52 | 7.47 ± 0.62 | 7.60 ± 1.17 |
| **HGB (g/dL)** | 15.40 ± 0.72 | 14.98 ± 0.62 | 14.80 ± 0.71 | 17.27 ± 2.94 |
| **HCT (%)** | 50.47 ± 1.76 | 48.90 ± 1.77 | 48.06 ± 2.63 | 51.12 ± 8.30 |
| **MCV (fL)** | 61.60 ± 1.8 | 63.58 ± 4.55 | 64.48 ± 1.87 | 67.33 ± 4.66 |
| **MCH (pg)** | 18.80 ± 0.79 | 19.48 ± 1.82 | 19.86 ± 0.70 | 22.70 ± 1.46 |
| **MCHC (g/dL)** | 30.50 ± 0.43 | 30.63 ± 0.84 | 30.80 ± 0.29 | 33.84 ± 2.68 |
| **RET (K/µL)** | 330.20 ± 50.22 | 482.58 ± 102.26 | 484.43 ± 97.90 | 265.93 ± 86.06 |
| **PLT (K/µL)** | 790.33 ± 153.08 | 836.50 ± 243.31 | 854.25 ± 28.37 | 1630.91 ± 405.29 |
| **WBC (K/µL)** | 12.26 ± 3.34 | 15.85 ± 6.09 | 12.98 ± 2.25 | 10.83 ± 3.84 |
| **NEUT (K/µL)** | 1.75 ± 0.81 | 1.26 ± 0.65 | 1.69 ± 0.41 | 3.31 ± 1.67 |
| **LYMPH (K/µL)** | 9.51 ± 2.40 | 10.85 ± 6.43 | 11.21 ± 2.12 | 6.72 ± 2.53 |
| **MONO (K/µL)** | 0.83 ± 0.37 | 0.69 ± 0.50 | 0.65 ± 0.26 | 0.67 ± 0.33 |
| **EO (K/ µL)** | 0.15 ± 0.07 | 0.13 ± 0.10 | 0.12 ± 0.10 | 0.13 ± 0.13 |
| **BASO (K/µL)** | 0.02 ± 0.01 | 0.03 ± 0.01 | 0.06 ± 0.04 | 0.03 ± 0.04 |
| **NEUT (%)** | 13.67 ± 3.51 | 9.85 ± 2.42 | 12.36 ± 2.17 | 14.80 ± 6.15 |
| **LYMPH (%)** | 78.03 ± 4.46 | 84.33 ± 3.23 | 79.90 ± 1.69 | 76.60 ± 4.96 |
| **MONO (%)** | 6.97 ± 2.68 | 4.80 ± 1.39 | 4.80 ± 1.99 | 4.86 ± 0.45 |
| **EO (%)** | 1.17 ± 0.37 | 0.83 ± 0.48 | 0.84 ± 0.59 | 3.60 ± 1.41 |
| **BASO (%)** | 0.17 ± 0.05 | 0.20 ± 0.08 | 0.38 ± 0.27 | 0.13 ± 0.05 |
| **RBC: red blood cell; HGB: hemoglobin; HCT: hematocrit; MCV: mean corpuscular volume: MCH: mean corpuscular hemoglobin; MCHC: mean corpuscular hemoglobin concentration; PLT: platelet; WBC: white blood cell; NEUT: neutrophil; LYMPH: lymphocyte; MONO: monocyte; EO: eosinophil; BASO: basophil.** | | | | |
